# Beyond the Chromosome: Recent Developments in Decoding the Significance of Extrachromosomal Circular DNA (eccDNA) in Human Malignancies

**DOI:** 10.3390/life14080922

**Published:** 2024-07-24

**Authors:** Panagiotis Tsiakanikas, Konstantina Athanasopoulou, Ioanna A. Darioti, Vasiliki Taxiarchoula Agiassoti, Stamatis Theocharis, Andreas Scorilas, Panagiotis G. Adamopoulos

**Affiliations:** 1 Department of Biochemistry and Molecular Biology, Faculty of Biology, National and Kapodistrian University of Athens, 15701 Athens, Greece; 2 First Department of Pathology, Medical School, National and Kapodistrian University of Athens, 15772 Athens, Greece; vasilikiagiassoti@gmail.com (V.T.A.);

**Keywords:** extrachromosomal circular DNA (eccDNA), circular DNA, eccDNA biogenesis, cancer, eccDNA detection, eccDNA biological role

## Abstract

Extrachromosomal circular DNA (eccDNA) is a form of a circular double-stranded DNA that exists independently of conventional chromosomes. eccDNA exhibits a broad and random distribution across eukaryotic cells and has been associated with tumor-related properties due to its ability to harbor the complete gene information of oncogenes. The complex and multifaceted mechanisms underlying eccDNA formation include pathways such as DNA damage repair, breakage–fusion–bridge (BFB) mechanisms, chromothripsis, and cell apoptosis. Of note, eccDNA plays a pivotal role in tumor development, genetic heterogeneity, and therapeutic resistance. The high copy number and transcriptional activity of oncogenes carried by eccDNA contribute to the accelerated growth of tumors. Notably, the amplification of oncogenes on eccDNA is implicated in the malignant progression of cancer cells. The improvement of high-throughput sequencing techniques has greatly enhanced our knowledge of eccDNA by allowing for a detailed examination of its genetic structures and functions. However, we still lack a comprehensive and efficient annotation for eccDNA, while challenges persist in the study and understanding of the functional role of eccDNA, emphasizing the need for the development of robust methodologies. The potential clinical applications of eccDNA, such as its role as a measurable biomarker or therapeutic target in diseases, particularly within the spectrum of human malignancies, is a promising field for future research. In conclusion, eccDNA represents a quite dynamic and multifunctional genetic entity with far-reaching implications in cancer pathogenesis and beyond. Further research is essential to unravel the molecular pathways of eccDNA formation, elucidate its functional roles, and explore its clinical applications. Addressing these aspects is crucial for advancing our understanding of genomic instability and developing novel strategies for tailored therapeutics, especially in cancer.

## 1. Introduction

The discovery of eccDNA can be traced back to 1964, when electron microscopy of wheat nuclei and boar sperm cells led to the detection of circular DNA outside chromosomes. A year later, paired structures known as double-minute chromosomes (DMs) were observed in metaphase spreads from bronchial carcinoma and neuroblastoma samples [1]. In 1981, functional studies highlighted the association between methotrexate resistance and dihydrofolate reductase gene amplification on DMs and homogeneous staining regions (HSRs) in the 3T6 mouse fibroblast cell line. In 1983, additional studies revealed the amplification of *MYCN* sequences in eccDNA within neuroblastoma cell lines, establishing a connection between eccDNA and oncogene amplification, particularly in the context of cancer development [2]. The progress made in understanding the structural and functional aspects of eccDNA has paved the way for contemporary investigations, especially with the advent of high-throughput sequencing technologies. These modern methodologies promise to unravel additional layers of complexity in the realm of extrachromosomal genetic material [3]. Nowadays, the study of eccDNA represents a promising frontier in modern cancer research, offering a novel perspective on the genomic landscape and providing deeper insights into the mechanisms driving tumorigenesis and cancer progression [4].

eccDNA stands out as a distinctive entity in the cellular landscape of eukaryotes. Replication errors, recombination events, and genomic instability are responsible for the dynamical generation of eccDNA and actively contribute to shaping the genomic architecture [5]. While the role of eccDNA in cancer has received significant attention, it is crucial to acknowledge its presence and potential functions in non-malignant cells. Recent studies suggest that eccDNA molecules are involved in normal cellular processes, including genomic plasticity, response to environmental stressors, and adaptation mechanisms. eccDNAs are found rarely and in low abundance in normal cells, although they can originate across the entire genome and are typically characterized by their small size (usually less than 3 Kb) [6]. In contrast, cancer-related eccDNAs display increased amplification and form large mega-base pair structures (>1 Mb) which contain numerous genes and regulatory regions [7]. In the context of cancer, eccDNA emerges as a critical player, contributing to genomic heterogeneity and therapeutic resistance [5,8]. The amplification of oncogenes on extrachromosomal elements has been linked to aggressive tumor phenotypes. Undoubtedly, the exploration of eccDNA in both malignant and non-malignant contexts will broaden our understanding of its biological significance not only in cancer pathogenesis but also beyond the cancer paradigm.

In this review, we provide a concise yet comprehensive overview of the biogenesis, structural formation, and biological role of eccDNA in both physiological and malignant cells. We discuss the cutting-edge technologies employed in studying eccDNA, offering a glimpse into the methods used for unraveling its mysteries. Finally, we explore the potential clinical applications of eccDNA in cancer, ranging from its role as a biomarker to therapeutic target, setting the stage for promising avenues in future cancer research and treatment.

## 2. eccDNA Biogenesis

**Figure 1:**
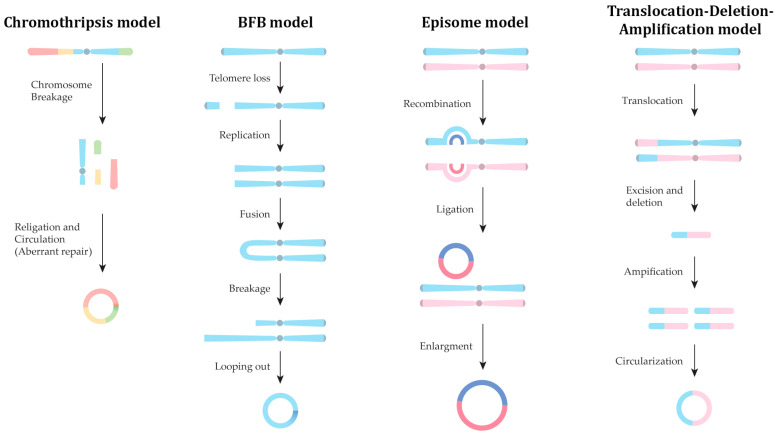
Overview of the major mechanisms implicated in eccDNA formation.

The elucidation of biogenetic pathways involved in eccDNA formation has recently become a point of intensive investigation, driven by their important role in shaping genomic architecture, contributing to genetic diversity, facilitating cellular adaption, and the fact that they have been implicated in disease etiology. Early indications suggest a context-dependent biogenetic process, with various cellular mechanisms participating in the generation of diverse eccDNA molecules. Biological processes like apoptosis, chromothripsis, and DNA damage repair are associated with eccDNA formation. Other proposed models include the episome model, the breakage–fusion–bridge cycle, and the translocation–deletion–amplification model, as well as fork stalling and template switching (Figure 1) [9].

### 2.1. DNA Damage Repair

In the standard model of eccDNA formation and maintenance, DNA repair mechanisms fail to properly repair DNA damage. From a mechanistic perspective, the generation of eccDNA requires the disruption of DNA integrity by environmental factors or internal cellular processes, followed by defective repair of DNA attributed to specific repair pathways. The study of eccDNA junctions provides insights into the potential repair mechanism involved in their formation. Erroneous activity of nonhomologous end-joining (NHEJ) and/or homologous recombination (HR), which are key DNA repair pathways, have been observed to lead to the formation of eccDNA [4,10,11,12,13]. Homologous recombination is proposed as a mechanism for eccDNA generation between repetitive DNA regions characterized by increased homology, although its contribution to the overall cellular eccDNA is limited due to its temporal restriction during mitosis [12,14]. Fragments resulting from double-strand breaks (DSBs) in DNA region homology primarily generate eccDNA through NHEJ [15]. This is supported by a simultaneous decrease in eccDNA abundance when proteins involved in the NHEJ repair mechanism are impaired [16,17]. Similarly, deficiencies in crucial genes involved in other repair mechanisms, such as DNA mismatch repair (MMR) and microhomology-mediated end-joining (MMEJ), have also been shown to decrease eccDNA amounts [18,19].

### 2.2. Chromothripsis and Apoptosis

While cancer development typically involves a gradual accumulation of genomic alterations, chromothripsis represents a single catastrophic genomic event where chromosomes undergo massive shattering. Various studies emphasize the pivotal role of DNA structural modifications in inducing the formation of eccDNA. Typically, DNA repair mechanisms eliminate DNA fragments; however, some fragments are randomly ligated or replicated, resulting in the formation of DMs [20,21,22]. Although malignant neoplasms display varying degrees of chromothripsis, approximately 2–3% of cancer samples display distinct genomic rearrangements attributed to this phenomenon. However, current research suggests an increased prevalence of chromothripsis events in cancer genomes [23]. The sequential impact of chromothripsis, followed by nonhomologous end-joining (NHEJ) and/or Poly(ADP-ribose)-dependent repair mechanisms, promotes oncogene amplification, drug resistance, and subsequent genomic rearrangements [21]. The chromothripsis model explains the formation of eccDNA carrying oncogenes, thereby facilitating tumor development and contributing to both genomic instability and DNA damage in cancer cells [24,25,26].

Programmed cell death or apoptosis is a form of a well-orchestrated cell death designed to eliminate unwanted or abnormal cells. The cleavage of chromosomal DNA is a well-documented hallmark of the apoptotic mechanism, mediated by enzymes such as caspase-activated DNase (CAD), endonuclease G, and DNase γ [27,28,29]. Similar to chromothripsis, apoptotic DNA fragmentation can induce the formation of eccDNA. Furthermore, pharmacologically induced apoptosis by chemotherapeutic agents results in a similar outcome [30]. It has been shown that during apoptosis, apoptotic DNA fragments are circularized by DNA ligase 3 (LIG3) to generate eccDNA [31]. These small eccDNA molecules (<1000 bp) exhibit high immunostimulatory activity, triggering the expression of type I interferon and/or potentially contributing to the cytokine storm observed in several diseases [30,31].

### 2.3. Breakage–Fusion–Bridge (BFB) Model

The breakage–fusion–bridge (BFB) model is a key mechanism that promotes gene amplification and confers instability upon the cancer genome. The BFB model was firstly described in 1951 using *Zea mays* as a model and later also observed in malignant human neoplasms [32,33,34]. The initial event of the BFB model is a DSB in a chromosome resulting in telomere loss. This loss of telomeres facilitates the fusion of chromosome ends, leading to the generation of a new dicentric chromosome. During anaphase, the dicentric chromosome forms a bridge as centromeres move apart in the kinetochore and finally breaks in a random position to produce new chromosome fragments that also lack telomeres. This incident leads to repeated cycles of the BFB model, promoting genomic instability, or even chormothripsis, while producing new eccDNA [35,36,37].

### 2.4. Episome Model

Episomes were initially identified in tumor samples as small circular DNA fragments known for their ability to replicate autonomously [4]. In the episome model, a DNA segment can be excised from a chromosome and rearranged into a circular DNA with a “head-to-tail” orientation. In other instances, episomes can arise by the collapse of stalling replication forks [38]. These episomes can expand into large eccDNA through self-replication and/or integration of other chromosomal regions through recombination [39]. The integration of cancer driver genes and regulatory DNA elements into large episomes has been observed in malignant cells, supporting the great importance of the episome model for the development of both solid tumors and hematological tumors [40,41,42].

### 2.5. Translocation–Deletion–Amplification Model

In this model, an external stimulus initiates chromosomal translocation, serving as the initial trigger. Chromosomal segments adjacent to translocation breakpoints can be excised and amplified, leading to the formation of amplicons. These amplicons can subsequently undergo circularization to form eccDNA, with proto-oncogene activation commonly associated with these events [43]. This phenomenon has been observed in neuroblastoma cell lines where a reciprocal translocation between chromosomes 8 and 16 [t(8;16)] promotes the amplification of proto-oncogene *MYC* and AT motif binding factor 1 (*ATBF1*) [43]. A similar effect has also been noted in the case of cancerous cells of carcinoma ex pleomorphic adenoma (Ca ex PA) where t(10;12) enables the amplification of *HMGIC* and *MDM2* [44].

## 3. Categorization and Classification of eccDNA

eccDNA can be categorized into five main categories considering its size, genomic origin, and sequence. These categories include large extrachromosomal DNA or DMs, telomeric circles, microDNA, and small polydispersed DNA (spcDNA).

### 3.1. Double-Minute Chromosomes (DMs)

DMs were the first type of eccDNA observed in cancer cells [45,46,47,48]. In general, DMs lack centromeres and telomeres, and their abundance varies significantly among cancer cells [49,50]. This variability can primarily be attributed to asymmetrical division, which results in frequent structural rearrangements. Consequently, these structures integrate multiple genomic regions from different loci [51]. Notably, in cancer cells, eccDNA is enriched with genomic regions harboring oncogenes and/or drug-resistance genes [25,52,53,54]. Alternatively, eccDNAs can serve as mobile regulatory elements, such as super enhancers, altering the transcriptional program of cancer cells. Generated by random genomic events, DMs provide cancer cells with an evolutionary advantage, thereby facilitating both tumor development and progression [51,55].

### 3.2. microDNA

microDNA has recently emerged as a novel type of circular DNA in eucaryotic cells with average length of 400 bp (ranging from 200 to 3000 bp) [56,57]. While the structure, size, and physical characteristics of microDNA resemble those of spcDNA, microDNA typically originates from active chromatin sites, incorporating exonic and 5′ and 3′ untranslated regions (UTRs) of genes, as well as GC-rich genomic regions compared to the whole genome [30,56]. Although microDNAs are relatively small in size and lack protein-coding ability, evidence suggests that they can be transcribed into several small, regulatory RNA molecules, such as microRNAs, which can regulate gene expression at the post-transcriptional level [57]. The formation and abundance of microDNA are directly impacted by DNA damage and the microhomology-mediated end-joining (MMEJ) repair mechanism [19]. microDNA is detected in both healthy and pathological conditions; however, the distribution, size, and abundance may vary in different types of biological samples. Of note, cancer cells can secrete microDNA into circulation, pointing towards a potential role as a biomarker for the noninvasive monitoring of disease progression and/or therapeutic efficacy [7,58].

### 3.3. Telomeric Circle (t-Circle/c-Circle)

Telomeric circles represent distinctive eccDNA entities that participate in the alternative lengthening of telomeres (ALT) in malignant tumors lacking telomerase activity, especially those of mesenchymal origin such as osteosarcoma and soft tissue sarcoma [59,60,61]. Telomeric circles are formed as double-stranded eccDNA containing telomeric repeats (t-circles) or characterized by a distinct single-stranded region rich in cytosines (c-circles) [62]. In the ALT mechanism, telomeric damage results in a telomeric DSB, which consequently serves as an entry point for telomeric circles to invade the damaged telomeric strand. Telomeric circles serve as a template in rolling cycle amplification (RCA) during telomere elongation [63,64].

### 3.4. Small Polydisperse Circular DNA (spcDNA)

spcDNA refers to the smallest fraction of eccDNA, ranging between 100 and 10,000 bp in size. Multiple cellular mechanisms including non-homologous recombination, HR, retrotransposition, and the DNA ligase IV-dependent pathway have been proposed to be involved in spcDNA generation [65,66,67,68]. It is characterized by diverse sizes, wide distribution, and integration of multiple genomic sequences [69]. It was first isolated in 1972 from the HeLa cell line [70]. Since then, it has been identified in various eukaryotic cells, with higher abundance observed in genetically unstable cells, linking it to increased genetic instability [71,72]. Genomic instability is mediated through autonomous replication of spcDNA and/or recombination events [73].

## 4. eccDNA Physiological Functions

**Figure 2:**
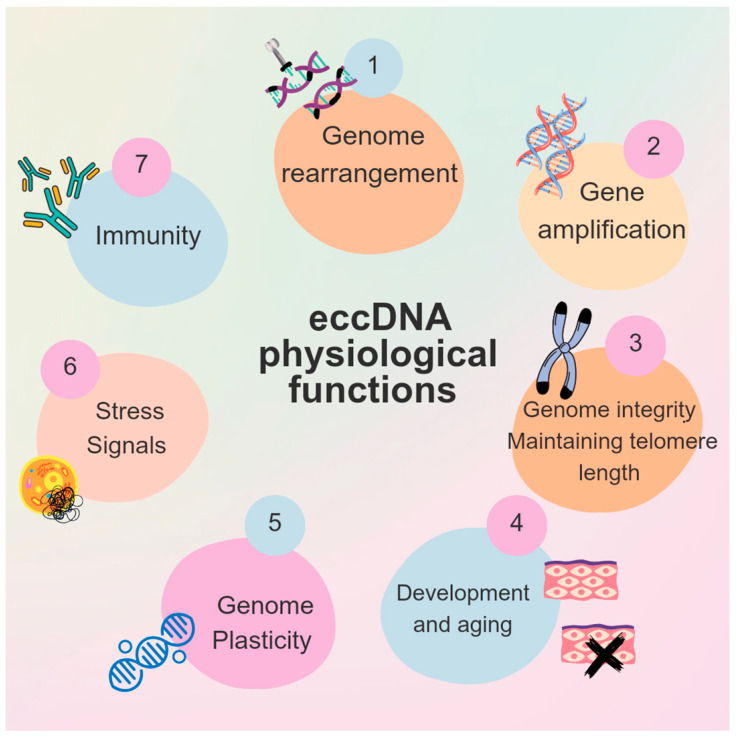
The physiological functions of eccDNA.

In recent years, an increasing number of studies have shed light on the various physiological processes in which eccDNA participates as a key regulatory element (Figure 2). Understanding its physiological role is essential for elucidating the contribution of eccDNA-dependent mechanisms to disease pathogenesis and progression. One well-established physiological role of eccDNA is the preservation of genome integrity by maintaining telomere length. Telomeric circles, including t-circles and c-circles, play a crucial role in ALT cells, serving as templates during telomeric DNA synthesis [61]. The accumulation of t-circles is associated with increased telomere trimming activity and enhanced cellular proliferation [74,75].

eccDNA has been observed in early developmental stages and is associated with aging in vertebrates. For example, telomeric circles have been identified during the early development of *Xenopus laevis* but are diminished in later stages and completely absent from adult tissues. This phenomenon contributes to increased embryonic genome plasticity, potentially playing a role in reshaping the genome during embryogenesis [76,77]. Several studies also highlight the association of eccDNA with aging, indicating a significant increase in eccDNA abundance in old cells of yeast and mammals [69,78]. The accumulation of eccDNA in mother yeast cells has been shown to promote aging [79]. Additionally, transcriptional reprogramming can stimulate eccDNA formation. The increase in genome plasticity through eccDNA formation under distinct transcription patterns is dictated by specific environmental conditions, representing a versatile strategy for yeast to adapt in their current environment [78].

To the best of our knowledge, there is limited information regarding the role of eccDNA in stem cell maintenance, differentiation, and aging. An increased amount of eccDNA can be observed in blood stem cells, which subsequently diminishes in terminally differentiated myeloid and lymphoid cells. This eccDNA represents mainly microDNA and is enriched in the 5’ UTR. Interestingly, more differentiated cells generally exhibit a higher abundance of eccDNA. Collectively, these findings underscore the significant role of eccDNA in normal hematopoiesis [6]. A recent study by Ren et al. found eccDNA in human stem cells, suggesting a potential role in the stem cell aging process [80]. The profiling of eccDNA in adipose stem cells from young and old individuals revealed eccDNA that was completely depleted in older individuals, identifying it as a potential target for stem cell rejuvenation. Although the absent eccDNA in older participants was small in size, incapable of being actively transcribed, all of it shared sequences from the *CAMK2G* and *TRABD2B* gene loci [80].

eccDNA serves as an alternative form of gene amplification by harboring multiple copies of genes and/or function as trans-acting super enhancers [50,55,81]. Consequently, eccDNA acts as an additional layer of regulation for multiple signaling pathways, guiding adaptive evolution and responses to environmental stimuli. A notable example is observed in yeast, where rapid adaptation to environmental changes is facilitated by alterations in gene copy numbers mediated by eccDNA [13,82,83]. Similarly, eccDNA has been implicated in the evolution of herbicide resistance in crop weeds following extensive use of glyphosate [84]. Moreover, oxidized eccDNA released by irradiated cells functions as a stress signal to bystander cells, sustaining the activation of oxidative signaling and finally promoting cell apoptosis [85,86].

Emerging evidence indicates that eccDNA can include sequences that are transcribed into regulatory RNAs such as microRNA (miRNA) and long non-coding RNA (lncRNA). microDNA constitutes a class of eccDNA that is incapable of encoding protein isoforms but is actively transcribed, even in the absence of canonical promoter sequences, into several regulatory RNA molecules [57]. Recently, it has been proposed that eccDNA generated by genome rearrangement can also be transcribed to produce rearrangement-specific non-coding RNAs (ncRNAs). However, the actual biological role of these transcripts remains to be unveiled by future studies [87].

eccDNA has the potential to act as an immunostimulant, activating the innate immune system response. In general, the induction of inflammation by circular DNAs has been observed in the case of mitochondrial DNA and heavily depends on its methylated CpG content and/or the presence of oxidatively damaged nucleotides [88]. This concept is further supported by studies on the activation of the innate immune system by eccDNA. eccDNA rich in CpG dinucleotides serves as a ligand for Toll-like receptor 9 (TLR9), leading to the activation of NF-κB signaling and increased expression of proinflammatory cytokines such as IL6 and TNFa [89,90]. Interestingly, the circularity of eccDNA is a key factor in its potency as an immunostimulant by activating the innate immune system response, which is mediated through the cGAS-STING pathway [31].

## 5. eccDNA’s role in Tumor Progression, Heterogeneity, and Evolution

eccDNA has been implicated in oncogene amplification, mutation acquisition, and the genomic instability of cancer cells [1]. eccDNA is prevalent in malignant cells, harbors oncogenic driver genes, and facilitates oncogene overexpression. Additionally, eccDNA can form clusters that function as mobile super-enhancers, further promoting proto-oncogene overexpression [50,91,92]. In cancer cells, focal amplification of multiple oncogenes, including *EGFR*, *ERBB2*, *MYC*, *BRAF*, *CDK4*, and *MDM2* is observed [50,93,94]. These oncogenes are among the most highly expressed across the cancer transcriptome [91]. The presence of these oncogenes enhances the “stemness”, self-renewal, and immunosuppressive microenviroment of cancer stem cell (CSCs) [95]. Moreover, comprehensive functional enrichment analyses of the genes carried by eccDNA in cancer cells revealed a significant enrichment in oncogenic pathways [96].

In addition to directly increasing the copy number of oncogenes, eccDNA can function as a trans-acting factor to regulate their expression [50,91,97]. Fluorescence microscopy has revealed that eccDNA molecules are organized into distinct structures known as eccDNA hubs. These hubs typically comprise 10–100 eccDNAs, encompassing the complete oncogene information and regulatory sequences. Their spatial proximity within these hubs is a critical factor in inducing oncogene overexpression. Accordingly, the disruption of eccDNA hubs through targeted genomic engineering approaches represses the transcription of oncogenes [91].

Unlike chromosomes, eccDNA does not adhere to conventional inheritance principles. Consequently, during mitosis of cancer cells, eccDNA is distributed unequally among daughter cells, providing a proliferation advantage to the corresponding cells, thereby enhancing tumor heterogeneity [98]. As eccDNA is randomly formatted in cancer cells and distributed to daughter cells, a great diversity of eccDNA is produced [25,99]. These molecules may not contain the complete genetic information of oncogenes and/or regulatory sequences, but they can function synergistically to increase tumor heterogeneity, providing cell-specific adaptions to external stimuli [91].

All these factors significantly increase the plasticity of the cancer genome and contribute to the adaptive evolution of cancer cells, profoundly impacting the treatment of malignant neoplasms. Cancer cells can develop resistance to targeted therapies through the involvement of eccDNA [100]. For example, in glioblastoma, cancer cells modulate expression levels of the oncogenic variant *EGFRvIII* to optimize growth and response to epidermal growth factor receptor tyrosine kinase inhibitors (EGFR-TKIs) [101]. While the presence of *EGFRvIII* eccDNA may not be entirely implicated in the adaptive selection of EGFR-TKI-resistant tumor cells, EGFRvIII can be rapidly eliminated following TKI administration, thereby increasing resistance in glioblastoma cells [16,101]. In melanoma, the BRAFV600E mutation drives progression to advanced stages of the disease. Focal amplification of BRAFV600E is associated with resistance to BRAF and MEK inhibitors. Pathogenic BRAFV600E variants are found in eccDNA and are amplified in response to drug administration [89].

## 6. Potential Clinical Applications of eccDNA in Cancer

eccDNA holds promise in cancer therapeutics due to its unique characteristics and potential impact on cancer cell biology. Aberrant levels of eccDNA have been observed in various solid tumors and hematological malignancies, suggesting its potential role as a biomarker for early detection, survival prediction, and therapy monitoring [6,39,102]. In patients suffering from neuroblastoma, eccDNA-driven chromosomal rearrangements of the *MYCN* oncogene have been associated with poor prognosis and overall survival compared to patients without the eccDNA-derived remodeling of *MYCN* [103]. Additionally, small eccDNA molecules, such as those originating from the tumor suppressor gene *MYO18B*, have been linked to cancer progression in lung and ovarian malignancies [104,105]. Similarly, the eccDNA generated by the prognostic biomarker *MEP1A* gene shows promise as a prognostic indicator in multiple cancer types [102,106,107]. In gastric cancer, *ERBB2* amplification on eccDNA has been associated with poor prognosis in ERBB2-positive patients [24]. Furthermore, high-throughput sequencing studies in breast cancer have revealed numerous eccDNA-related differentially expressed genes, including *CTNNB1*, *CACNA2D2*, and *CACNA1D*, which are implicated in breast invasive carcinoma tissues [108]. In acute myeloid leukemia, ATAC-seq experiments have detected eccDNAs, indicating their prognostic value regarding disease aggressiveness and patient survival outcomes. For instance, the poor prognostic factor *GLYATL1* gene, which is associated with eccDNA, has been found highly expressed in leukemia cells [6].

The direct targeting of the oncogenes amplified on eccDNA might offer a novel therapeutic strategy for cancer treatment in various human malignancies (Table 1). In glioblastoma, *EGFR* and *PDGFRA* amplification are responsible for extrachromosomal mutations [101]. Thus, disrupting the replication, maintenance, or stability of eccDNA may impair cancer cell viability or sensitize cancer cells to existing treatments. An additional example involves methotrexate, which is an inhibitor of dihydrofolate reductase and serves as an antitumor factor in most malignant tissues. However, in colorectal cancer, cells are resistant to methotrexate due to the amplification of *DHFR* by eccDNA [92]. Depletion of the DNA-PKc protein, which is related to NHEJ in eccDNA, reduces DHFR levels in colorectal cells, overcoming methotrexate resistance, indicating that the NHEJ pathway constitutes a target for cancer treatment [16].

eccDNA often harbors oncogenes, tumor suppressor genes, or other cancer-associated genetic alterations. Characterizing the eccDNA primary sequence may facilitate the identification of driver mutations responsible for cancer development and progression, potentially guiding therapy selection. In liquid biopsy samples, circulating tumor DNA (ctDNA) derived from tumor cells may contain eccDNA fragments carrying *EGFR* mutations or amplifications. eccDNA detected in bodily fluids, such as blood or urine, could enable non-invasive monitoring of cancer progression and treatment response [109]. Targeting eccDNA-related pathways in drug development may lead to the discovery of novel anticancer agents. Screening for agents specialized in disrupting eccDNA dynamics can foster the development of novel therapeutic strategies for cancer treatment [110]. Exploiting eccDNA biology for the targeted delivery of therapeutic genes or gene editing tools holds promise for cancer gene therapy. eccDNA-based vectors could offer advantages such as high transfection efficiency and prolonged transgene expression. Alterations in eccDNA abundance or structural characteristics may serve as early indicators of treatment efficacy or resistance, guiding therapeutic decisions. Specific eccDNA signatures may predict response to treatment, contributing to personalized treatment and reducing the severe impact of ineffective and potentially harmful treatments.

## 7. Detection Methods

Detecting and characterizing eccDNA pose significant challenges due to its heterogeneous nature, low abundance, and dynamic properties within cells. The study of eccDNA involves two main strategies: the identification of eccDNA by using sequencing-based approaches and by imaging and visualization methods. Sequencing-based approaches harness the power of next-generation sequencing (NGS) technologies to analyze DNA libraries and identify circular DNA molecules. Microscopy-based approaches provide spatial and temporal information about eccDNA localization and dynamics within cells [111,112]. A summarizing overview of the described techniques is provided in Table 2.

### 7.1. Sequencing-Based Approaches

The most common NGS application employed in the study of eccDNA includes Whole-Genome Sequencing (WGS) and Targeted DNA Sequencing. WGS enables sequencing of the entire genome, including both chromosomal DNA and extrachromosomal elements. eccDNA is captured along with linear genomic DNA fragments, and bioinformatics analysis is performed to identify eccDNA based on its unique read patterns, which are indicative of circularization events. WGS provides a detailed overview of the eccDNA landscape across the genome, revealing its abundance and size distribution [113].

Targeted DNA sequencing strategies reveal the primary structure of specific genomic regions and provide increased accuracy for the detection of the eccDNA, sequence content, and their potential functional significance. However, the short sequencing reads generated are limited in their ability to identify small eccDNA. Additionally, inverse PCR, circle PCR, and rolling-circle amplification (RCA) are used to selectively amplify circular DNA molecules [114]. Specific primers, designed to target circular DNA junctions or other characteristic sequences, are used and the amplified products are sequenced. Conventional biochemical methods, combined with sequencing, constitute complementary strategies for isolating and characterizing eccDNA in a relatively pure form from complex cellular extracts. In specific, cesium chloride (CsCl) density gradient centrifugation is used to separate DNA molecules based on their buoyant density. In CsCl gradients, eccDNA forms distinct bands at characteristic densities; thus, it can be purified from other DNA species and sequenced. However, the method requires a huge amount of starting material and obtains a low yield of eccDNA [35,115].

While the Assay for Transposase-Accessible Chromatin sequencing (ATAC-seq) is primarily designed to assess chromatin accessibility in the nuclear genome, it can be applied to indirectly identify certain types of eccDNA. ATAC-seq is based on the principle of transposase-mediated “tagmentation”, where a hyperactive Tn5 transposase simultaneously fragments and tags open chromatin regions with sequencing adapters. eccDNA within the nucleus, which is accessible to the transposase enzyme during the tagmentation step, can potentially be tagged with sequencing adapters and subsequently sequenced along with genomic DNA fragments and detected in ATAC-seq data as additional peaks or signals compared to background levels. However, factors such as the localization of eccDNA within the nucleus and the potential inefficiency in capturing low-abundance eccDNA during the tagmentation step can impact the detection sensitivity and accuracy of eccDNA in ATAC-seq data [116]. Chromatin Immunoprecipitation Sequencing (ChIP-Seq) has been applied to provide insights into protein binding to eccDNA and the localization of eccDNA in chromatin. An essential consideration is that the detection of eccDNA in ChIP-seq data hinges on the specificity of the antibody used during immunoprecipitation [5].

Although ChIP-seq is a powerful technique, two recently developed techniques that integrate chromatin conformation capture with ChIP-seq have emerged to provide additional insights into chromatin architecture and protein–DNA interactions, namely PLAC-seq (Proximity Ligation-Assisted ChIP-seq) and HiChIP (High-throughput Chromatin Conformation Capture with Immunoprecipitation). PLAC-seq combines chromatin immunoprecipitation with proximity ligation to enable the mapping of long-range chromatin interactions mediated by a protein of interest and can also be used to identify interactions between eccDNA and proteins [117]. DNA fragments originating from eccDNA, bound to the protein of interest, can undergo ligation with genomic DNA fragments during proximity ligation and then be sequenced subsequently [118,119]. On the other hand, HiChIP combines chromatin conformation capture with chromatin immunoprecipitation to investigate the spatial organization of chromatin and protein–DNA interactions, simultaneously, but can also be applied for the study of eccDNA by capturing DNA fragments originating from eccDNA [120]. Notably, both PLAC-seq and HiChIP are not specifically designed to study eccDNA; hence, the detection of eccDNA may be limited by factors such as the specificity of the antibody and the efficiency of proximity ligation [121].

CIDER-Seq, which stands for “Circular DNA Enrichment sequencing”, is widely used for eccDNA characterization from complex genomic samples. CIDER-Seq involves an initial enrichment step through exonuclease digestion to selectively degrade linear DNA or restriction enzyme digestion to target specific circular DNA structures. Sequencing adapters are ligated to the ends of circular DNA molecules, allowing for subsequent amplification and sequencing. By selectively amplifying circular DNA, CIDER-Seq reduces the background noise associated with linear genomic DNA fragments, thus enhancing the sensitivity and specificity of eccDNA detection compared to traditional sequencing approaches [122]. Despite its advantages, validation of CIDER-Seq results using orthogonal methods, such as PCR, is often necessary. In the same manner, Circle-Seq also involves a sensitive step to capture circular DNA from genomic DNA samples using exonucleases or restriction enzymes, followed by DNA library construction and high-throughput sequencing [13,123].

4C-seq (Circular Chromosome Conformation Capture Sequencing) is a derivative of the 3C method, specifically designed to investigate the spatial interactions of a selected genomic locus with other regions of the genome. In the context of studying eccDNA, cross-linked chromatin is digested with a restriction enzyme, and then subjected to proximity ligation to form chimeric DNA fragments. Inverse PCR is then used to amplify DNA fragments that are ligated to the selected genomic locus of interest and sequencing is performed in NGS platforms [124]. While 4C-seq may not be specifically optimized for studying eccDNA, it can still offer valuable information about the spatial relationships between eccDNA and chromosomal DNA [125].

In MNase-seq (Micrococcal Nuclease Sequencing), chromatin is treated with MNase endonuclease to digest the linker DNA, leaving behind nucleosome-protected DNA fragments. After digestion, the released DNA fragments are subjected to library preparation. Sequencing adapters are ligated to the ends of the DNA fragments, allowing for subsequent amplification and sequencing. EccDNA may exhibit unique nucleosome occupancy profiles compared to chromosomal DNA, reflecting its distinct chromatin organization. Thus, changes in chromatin accessibility detected by MNase-seq may indicate the presence of eccDNA and can reveal nucleosome positioning patterns associated with eccDNA [126,127]. EccDNA-derived signals in MNase-seq data may be influenced by factors such as sequence composition, chromatin context, and the efficiency of MNase digestion.

Although a plethora of NGS-based workflows have been developed to accurately identify eccDNA molecules, NGS still possesses some key limitations compared to the cutting-edge Third-Generation Sequencing (TGS) technology. Since PacBio sequencing performs real-time sequencing at a single-cell level, SMOOTH-seq (single-molecule real-time sequencing of long fragments amplified through transposon insertion) has been employed to detect eccDNA and enables functional studies within individual cells [128]. On the other hand, nanopore sequencing technology enables long sequencing reads, avoiding both fragmentation steps during sample preparation and the assembly of the sequencing reads during bioinformatics analysis. While nanopore sequencing is primarily used for WGS, transcriptome sequencing, and metagenomics, it can also be applied to study eccDNA. Nanopore sequencing offers real-time sequencing capabilities, allowing for the rapid detection and analysis of eccDNA without the need for complex library preparation or amplification steps, avoiding the introduction of biases and errors. Nanopore sequencing can detect eccDNA molecules directly by sequencing DNA fragments extracted from cellular or tissue samples [129,130], and can also provide quantitative information about the abundance of eccDNA molecules in a sample. Integration of nanopore sequencing with other experimental techniques can enhance our understanding of eccDNA biology and open new avenues into the exploration of eccDNA's role in cells [131].

### 7.2. Image-Based Approaches

Numerous microscopy-based methods, including FISH and transmission electron microscopy (TEM), have been employed to provide novel insights into eccDNA dynamics, replication, segregation, and stability within living cells. Moreover, over the last decade CRISPR-based methods have been developed to track eccDNA in real time. High-resolution microscope approaches enable the visualization of eccDNA and are often employed to validate eccDNA identified via sequencing-based approaches. However, many limitations arise from the sample availability. For instance, eccDNA can be observed in specific cell cycle phases, whereas some eccDNA signals are hardly detectable. On the other hand, transmission electron microscopy (TEM) and scanning electron microscopy (SEM) offer ultra-high resolution imaging capabilities suitable for studying small eccDNA structures [114].

FISH is used to study the distribution of eccDNA in living cells, by utilizing labeled DNA probes which are hybridized on target regions of eccDNA in metaphase cells. Although the method effectively distinguishes and quantifies the eccDNA-related signals from chromosomal DNA signals, it suffers from limited throughput. Moreover, DAPI (4′,6-diamidino-2-phenylindole) is a fluorescent DNA-binding dye which can be utilized in conjunction with FISH to quantify eccDNA within cells. Up to date, two widely used image analysis tools have been applied to accurately resolve eccDNA. ECdetect constitutes a semi-automated image analysis software package used for the quantification of eccDNA within the chromosomal landscape by analyzing the fluorescent signals from DAPI-stained cells [25,132]. While ECdetect accurately segmented intact nuclei and chromosomes, it could not automatically differentiate them. This limitation hindered its ability to identify FISH-stained homogeneously staining regions, which are indicative of gene amplification. On the contrary, ecSeq can automatically classify DAPI-stained metaphase images into the chromosome and eccDNA classes and combines DAPI signal classification, eccDNA identification, and FISH data integration to unravel the complexities of genomic architecture [133].

Nowadays, CRISPR (Clustered Regularly Interspaced Short Palindromic Repeats) systems serve as revolutionary engineering tools that allow precise genome editing. By leveraging the programmable nature of CRISPR/Cas systems, researchers can develop innovative methods for studying eccDNA in various biological contexts. One approach involves fluorescent reporters to visualize eccDNA in live cells. CRISPR/Cas is used to visualize the movement and behavior of eccDNA in living cells, by coupling the inactive form of Cas9 with fluorescent molecules and directing it to DNA breakpoint junctions on eccDNA. For instance, ecTag has been developed to visualize the spatiotemporal dynamics of eccDNA contributing to intra-tumoral heterogeneity and is based on labeling eccDNA elements with multiple fluorescent molecules [134]. An additional approach, namely CRISPR FISHer, combines CRISPR-based genome editing with FISH to track the movement of small eccDNA. CRISPR FISHer provides valuable insights into the behavior and dynamics of eccDNA, shedding light on its roles in cellular processes and potentially revealing its involvement in disease states or cellular abnormalities [135]. Alternatively, CRISPR/Cas systems specifically target sequences unique to eccDNA to induce cleavage or modification of eccDNA upon binding. After treatment with CRISPR/Cas complexes, genomic DNA can be extracted from cells and subjected to analysis methods, such as PCR, sequencing, or Southern blotting. For instance, the CRISPR-CATCH method utilizes the CRISPR/Cas system to isolate eccDNA molecules that can be detected by sequencing methods, including nanopore sequencing [136].

## 8. Conclusions

The study of eccDNA holds significant promise for advancing our understanding of heredity, evolution, and disease, especially in oncology. eccDNA, a unique form of genetic material, enables rapid cellular adaptation and evolution. Despite recent advancements, eccDNA research is still in its early stages, presenting both exciting opportunities and substantial challenges. Several mechanisms have been described in eccDNA biogenesis, including damage repair pathways, the BFB cycle, and chromothripsis. The most prominent mechanisms involve both massive chromosomal breakage and mild DNA damage to produce linear fragments that reassemble randomly into eccDNA through HR and NHEJ repair mechanisms, or alternatively, the loss of telomeres initiating multiple BFB cycles.

In oncology, eccDNA contributes to tumor instability, drug resistance, and heterogeneity by promoting gene amplification, transcriptional regulation, oncogene rearrangement, and intercellular signaling. Under that prism, it holds promise as a biomarker in liquid biopsies, potentially improving the accuracy of disease diagnosis, prognosis, and treatment selection. However, current detection methods need refinement to increase sensitivity and exclude artifacts for reliable clinical application. Advanced microscopy- and sequencing-based techniques are essential for distinguishing eccDNA from chromosomal DNA and characterizing its structure and function. Developing even more sensitive detection tools will support eccDNA research and clinical applications. Future studies should focus on understanding the chromatin structure and sequence characteristics of eccDNA, identifying key regulators and interactions even at the single-cell level. These efforts will elucidate its role in cancer development and other biological processes. Moreover, that eccDNA is involved in gene amplification and drug resistance highlights its potential as a therapeutic target. Therapeutic interventions targeting eccDNA could regulate gene expression and enhance drug sensitivity, offering novel approaches for cancer treatment.

In conclusion, eccDNA research could revolutionize our understanding of genetic material and its role in disease, particularly cancer. By addressing current challenges and leveraging advanced technologies, researchers can unlock eccDNA’s full potential, paving the way for innovative diagnostic and therapeutic strategies. Encouraging further studies in this field is crucial for realizing the significant promise that eccDNA holds for improving health outcomes.

## Figures and Tables

**Table 1:** Genes identified on eccDNA in human malignant cell lines.

Human Cancer	Sample Type	Protein-Coding Genes Found on eccDNA
Acute myeloid leukemia	HL-60	*MYC*
Breast cancer	T-47D	*C16orf74*, *EMC8*, *GINS2*, *GSE1*
	BT-474	*ADA*, *ANKRD60*, *APCDD1L*, *AURKA*, *BMP7*, *C20orf85*, *CASS4*, *CBLN4*, *CCN5*, *CSTF1*, *CTCFL*, *FAM209A*, *FAM209B*, *FAM210B*, *GCNT7*, *HNF4A*, *KCNK15*, *MC3R*, *MTRNR2L3*, *NPEPL1*, *PABPC1L*, *PCK1*, *PI3*, *PKIG*, *PMEPA1*, *RAB22A*, *RAE1*, *RBM38*, *RIMS4*, *RTF2*, *SEMG1*, *SEMG2*, *SERINC3*, *SPO11*, *STK4*, *STX16*, *STX16-NPEPL1*, *TFAP2C*, *TOMM34*, *TTPAL*, *VAPB*, *WFDC12*, *WFDC5*, *ZBP1*
	HCC1569	*C19orf12*, *CCNE1*, *ERBB2*, *GRB7*, *IKZF3*, *PGAP3*, *PLEKHF1*, *POP4*, *UQCRFS1*, *URI1*, *VSTM2B*, *ZNF536*
	MCF-7	*ARFGEF2*, *CADPS*, *EYA2*, *INTS2*, *MED13*, *NCOA3*, *PREX1*, *RTEL1*, *STMN3*, *SULF2*, *SYNPR*, *ZMYND8*
Chronic myelogenous leukemia	K-562	*GPC5*
Colorectal cancer	SW-620	*MYC*
Glioblastoma	GBM6	*C1QTNF9*, *EGFR*, *PCDH9*, *SPATA13*
	BT-70	*ASAP1*, *CYRIB*
	GBM39	*EGFR*, *LANCL2*, *MRPS17*, *NIPSNAP2*, *PSPH*, *SEPTIN14*, *ZNF713*
	SF268	*ABCG4*, *ARCN1*, *ATP5MG*, *BCL9L*, *C1QTNF5*, *C2CD2L*, *CBL*, *CCDC153*, *CENATAC*, *CNTN5*, *CXCR5 NLRX1*, *DDX6*, *DPAGT1*, *FOXR1*, *GRIA4*, *HINFP*, *HMBS*, *HYOU1*, *IFT46*, *KMT2A*, *MCAM*, *MFRP*, *MSANTD4*, *NECTIN1*, *PDZD3*, *PHLDB1*, *RNF26*, *SLC37A4*, *THY1*, *TMEM25*, *TREH*, *TTC36*, *UPK2*, *USP2*, *VPS11*
	CA718	*CHIC2*, *CPM*, *DDX1*, *GSX2*, *LNX1*, *MDM2*, *MYCN*, *PDGFRA*, *SLC35E3*
	HK423	*COBL*, *EGFR*, *LANCL2*, *VOPP1*
	HK359	*ANKIB1*, *EGFR*, *GSTK1*, *KRIT1*, *PTPRN2*, *SEPTIN14*, *TMEM139*
	HK296	*ATP2B4*, *EGFR*, *ETNK2*, *GOLT1A*, *KISS1*, *LAX1*, *LRRN2*, *MDM4*, *NFASC*, *PIK3C2B*, *PLEKHA6*, *PPP1R15B*, *REN*, *SNRPE*, *SOX13*, *ZBED6*, *ZC3H11A*
Lung cancer	H460	*MYC*
	H23	*POU5F1B*
	H522	*CLPS*, *CLPSL1*, *LHFPL5*
	HCC827	*EGFR*, *LANCL2*, *SEC61G*, *VOPP1*
	EKVX	*KCNN3*, *PPM1F*, *TOP3B*
Medulloblastoma	RCMB20	*ADGRB1*, *ASAP1*, *CYRIB*, *FAM135B*, *KHDRBS3*, *MYC*, *POU5F1B*, *TSNARE1*, *ZFAT*
	MB211FH	*MYC*
	MDT-MB 1377	*CYRIB*, *MYC*
	MDT-MB 0007	*C1QL2*, *C2orf76*, *CFAP221*, *DBI*, *EN1*, *EPB41L5*, *GLI2*, *GPC5*, *INSIG2*, *MARCO*, *PTPN4*, *SCTR*, *STEAP3*, *TFCP2L1*, *TMEM37*
	MB002	*MYC*, *POU5F1B*
Ovarian cancer	OVCAR-3	*KRT18P4*, *SNAI1*, *SNRPFP1*
Prostate cancer	PC-3	*COLEC10*, *FAM135B*, *KCNK9*, *RPS26P35*, *SAMD12*, *TNFRSF11B*
Renal cancer	SN12C	*KANSL1*

**Table 2:** Overview of sequencing- and image-based methodologies, common methods used for the study of eccDNA.

Sequencing-Based Approaches	
Approach	Description
WGS	Capturing eccDNA with linear fragments of gDNA
Targeted DNA seq	
CRISPR-CATCH	Isolation of eccDNA and sequencing
ATAC-seq	Indirect identification of certain types of eccDNA
ChIP-seq	Identification of eccDNA located on chromatin and proteins binding to eccDNA
PLAC-seq	Identification of interactions between eccDNA and proteins
HiChIP	Capturing of eccDNA fragments to study chromatin organization and protein–DNA interactions
CiDER-seq	Selective amplification of eccDNA for enhancing sensitivity of detection
4C-seq	Detection of spatial relationships between eccDNA and chromosomal DNA
MNase	Characterization of nucleosome positioning patterns associated with eccDNA
Smooth-seq	Real-time detection of eccDNA at a single-cell level
Nanopore sequencing	Real-time sequencing for rapid detection of full-length eccDNA
**Image-based approaches**	
**Approach**	**Description**
FISH	Detection and quantification of eccDNA-related signals from chromosomal DNA
Ecdetect	Segmentation of intact nuclei and chromosomes
EcSeq	Automatic classification of DAPI-stained metaphase images of the chromosome
High-resolution microscope	Visualization of eccDNA
TEM	Ultra-high resolution imaging for the detection of small eccDNA
SEM	Ultra-high resolution imaging for the detection of small eccDNA

## Data Availability

No new data were created or analyzed in this study. Data sharing is not applicable to this article.
